# A molecular dynamics study on anticorrosion properties of graphene/epoxy nanocomposites in NaCl medium

**DOI:** 10.1039/d5ra08572a

**Published:** 2026-07-03

**Authors:** Jae-Hyok Ju, Ryong-Chol Ri, Il-Chol Ju, Chol-Nam Song, Ryong-Hyon Kong, Jae-Gwang Ri

**Affiliations:** a Branch Academy of Nano Engineering, Jonjin-Dong Rakrang District Pyongyang Democratic People's Republic of Korea cjh1983@star-co.net.kp

## Abstract

This work aims evaluate the role of graphene derivatives in epoxy coatings. We proposed new models composed of iron, graphene derivative/epoxy composites and a corrosion medium (NaCl), and evaluated the effects of the graphene derivatives in the epoxy coatings on the corrosion of iron by using Molecular Dynamics (MD) and Density Functional Theory (DFT). We used graphene derivatives such as pristine graphene (PG), graphene oxide (GO), reduced graphene oxide (RGO), and functionalized graphene oxide (FGO, where F represents two forms of nitrogen-based groups, which are amine –NH_2_ and amide –C(=O)–NH_2_), and also modeled pure epoxy coatings for comparison. The interaction energy between the graphene derivatives and epoxy resin increased in the order of PG, GO, RGO and FGO, and the interaction energy between the epoxy coating layer and the iron layer was rather larger than that of coatings with graphene derivatives. Interestingly, during the simulation, the interaction energy between the pure epoxy coating and the iron was reduced compared to that of the epoxy coating with GO, RGO and FGO. The wear of the coatings during the simulation was low for the coatings with PG and FGO, although the coatings with GO and RGO showed more severe wear than the pure epoxy coating. Finally, the permeation coefficient of the coatings was calculated. It was largest with PG, and reduced in the order of RGO, FGO and GO in the composite epoxy coatings. The results showed graphene derivative/epoxy composites had significantly better corrosion resistance than pure epoxy; in particular, the epoxy resin composite with GO represented the best corrosion resistance. This study may be important for developing graphene-reinforced plastic composites with good anticorrosion properties.

## Abstract 2

We report the corrosion resistance of graphene derivatives in epoxy coatings, studied *via* molecular dynamics. The graphene derivative/epoxy composites showed significantly better corrosion resistance than pure epoxy, especially the composite with GO.

## Graphical abstract

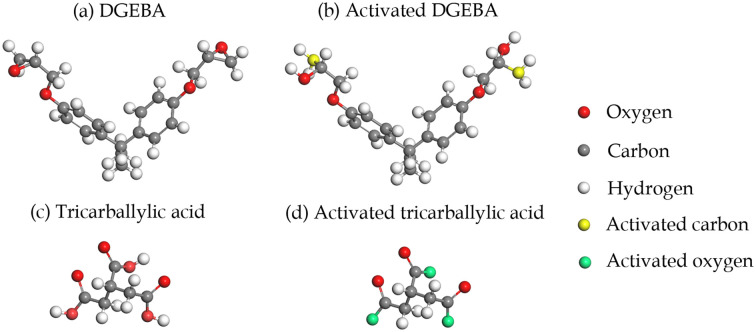

## 1 Introduction

Corrosion is the main cause of deterioration of the safety and durability of metal structures in industry.^1–7^ Corrosion of metals is a very complex process that is affected by various factors such as purity of metals, composition of the medium, temperature, salinity, and atmospheric humidity. Since corrosion causes huge economic losses, much attention is paid to the study of corrosion prevention.^1–3,8^

There are some methods to prevent corrosion, such as selection of suitable materials, environmental changes, proper design, electrochemical protection, and coating.^9^ There are also methods to change the properties of a metal, such as coating, surface passivation, and coating with refractory compounds. Although painting also plays a role in corrosion prevention, its efficiency is low, so there is a need to research materials with better corrosion resistance.^5^

Organic coatings are a physical interface between the corrosion medium and the metal; it is an effective solution for protecting the substrate,^2,7,10,11^ and epoxy coatings are an example. Epoxy resin has superior properties, such as strong adhesion, anticorrosion resistance, and low curing shrinkage, so it has been assessed a suitable material.^1,6,12–18^

Even with a coating of epoxy resin, hydrophilic groups and voids exist on the coating surface, so corrosive agents may penetrate into the metal/coating contact surface.^15^ Therefore, to overcome these drawbacks, much research has been carried out to improve corrosion resistance using a method that combines inhibitors with paint.^1–5,10–12,19–25^ Nanosized fillers with large specific surface area have been investigated to eliminate defects in the coating and to enhance the crosslinking density.^26^

Among nanofillers, graphene derivatives have superior properties, such as good mechanical and electrical properties, thermal stability, and barrier effects, so they have been widely studied for coating applications.^5–7,14,15,27–33^ The special electrical properties of graphene may also prevent metal corrosion by preventing reactions on the surface of the metal caused by electron transfer from the anode of the local corrosion cell formed on the metal surface beneath the polymer paint to the surface of the coating.^34^ Moreover, a graphene composite paint improves the overall properties of paint by combining the strong adhesion properties of graphene with the film-forming properties of the coating substrate.^35^

Many studies have been performed for development and application of graphene composite paints,^5^ and also to theoretically verify the anticorrosion properties *via* simulation. Computer simulation is a very powerful tool in solving problems that are difficult to solve using theory and experiment, and it is very important to analyze the open problems and simulate experiments.^36^

To theoretically simulate the anticorrosion process, researchers have used Density Functional Theory (DFT)^1,2,4,5,10–12,20–22,24,25,27,29–32,34^ and Molecular Dynamics (MD).^2–4,10–12,20–22,24,34^ Using DFT, they have calculated the HOMO, LUMO and dipole moments, electronic affinity, ionization potential, and electronegativity of inhibitors and indirectly evaluated the corrosion inhibition effect of inhibitors. Using MD, they have made models consisting of a substrate and corrosion medium, added the inhibitor and calculated the interaction energy between the substrate and inhibitor. Haiyan Wang *et al.* have established a system of inhibitor and corrosion media and evaluated the corrosion resistance by simulating the self-diffusion coefficient of the corrosion media *via* molecular dynamics methods.^19^

Studies on the improvement of mechanical properties of nanocomposites using molecular dynamics simulations of graphene-reinforced epoxy composites have been reported.^37–40^ Dikshit *et al.* carried out studies with 5, 10, 15 and 25 wt% graphene content,^38^ Mahmud *et al.* carried out studies with 19.6 wt% pristine graphene (PG), 31.5 wt% graphene oxide (GO), 29.5 wt% functionalized graphene oxide (FGO)^39^ and Faragi *et al.* carried out studies with 4.6, 8.8, 12.6, 16.1, and 19.4 wt% graphene.^40^ In practice, the price of graphene is so high that the quantity of graphene used in coatings is small, and it is difficult to consider these models as practical ones for nanocomposites.

To evaluate the corrosion resistance of graphene/epoxy composites with high accuracy, a system should be set up with a protected metal, graphene/epoxy coating (where the graphene should be added in a suitable quantity), and corrosion medium, but simulations for such a system have never been performed. In this study, we modeled a hybrid system with a protected metal and graphene derivative/epoxy coating (with the content of graphene set at a practical quantity), and evaluated the corrosion resistance *via* MD.

We used graphene derivatives such as PG, GO, reduced graphene oxide (RGO) and FGO, and also carried out modeling with pure epoxy coatings for comparison. The anticorrosion properties were evaluated by simulating the interaction between the graphene derivative and epoxy resin, the interaction between the substrate (iron) and the coating, the wear properties of the coating and the permeation coefficient.

## 2 Modeling and methods

We used LAMMPS 2022, Materials Studio 2023 for modeling and simulation, and PCFF (polymer consistent force field) in LAMMPS.^41^

All the molecules used in the modeling (DGEBA, tricarballylic acid, graphene derivatives, and water) were geometrically optimized *via* DFT (B3LYP, 6-311G∗*) before modeling. To make the atomic configuration energy-minimized and equilibrated, all equilibration was performed with a time step of 1 fs for 5 ns using the NPT ensemble (298.0 K, atmospheric pressure), and subsequently at 298.0 K using NVT ensembles. We used the Verlet algorithm for numerical integration of the equation of motion and the Nosé–Hoover thermostat and barostat.

### 2.1 Crosslinking of epoxy resin

**Fig. 1:**
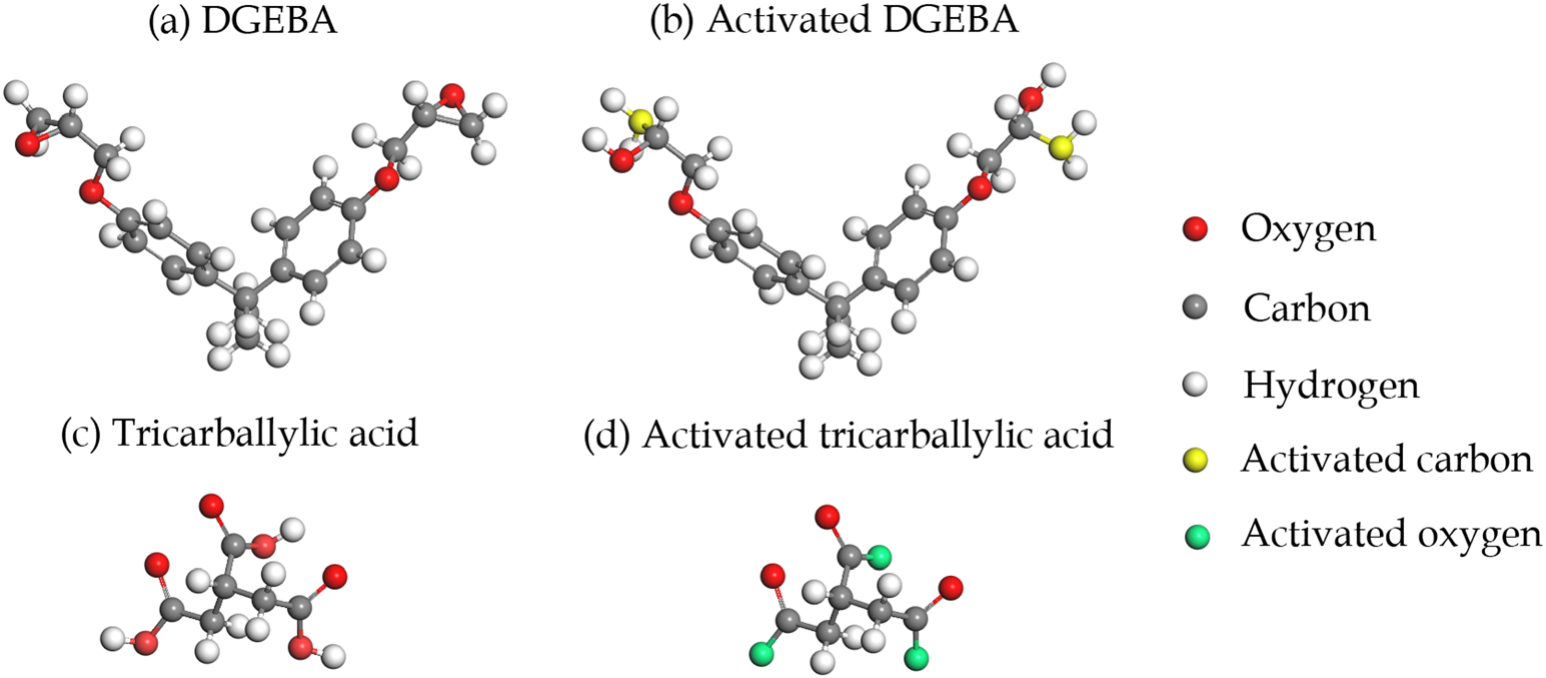
Structure of epoxy DGEBA and cross-linking agent molecules (a: DGEBA, b: activated DGEBA, c: tricarballylic acid, d: activated tricarballylic acid).

The epoxy resin was modeled as a network of DGEBA cross-linked by tricarballylic acid (crosslinking agent). For crosslinking, the epoxy and the crosslinking agent molecules were first activated. The structures of the monomer, crosslinking agent, and activated monomer and crosslinking agent for the crosslinking of the epoxy resin are shown in Fig. 1.

The epoxy coating was modeled using the Amorphous Cell module with an activated epoxy molecule and a crosslinking-agent molecule. The cell size was 37.142445 × 37.142445 × 50.0 Å^3^, the number of active sites of DGEBA was 2, and the number of active sites of tricarballylic acid was 3. Thus, we created an amorphous cell whose molecular number ratio was 3 : 2 (DGEBA : tricarballylic acid) and density was 1.16 g cm^−3^.

**Fig. 2:**
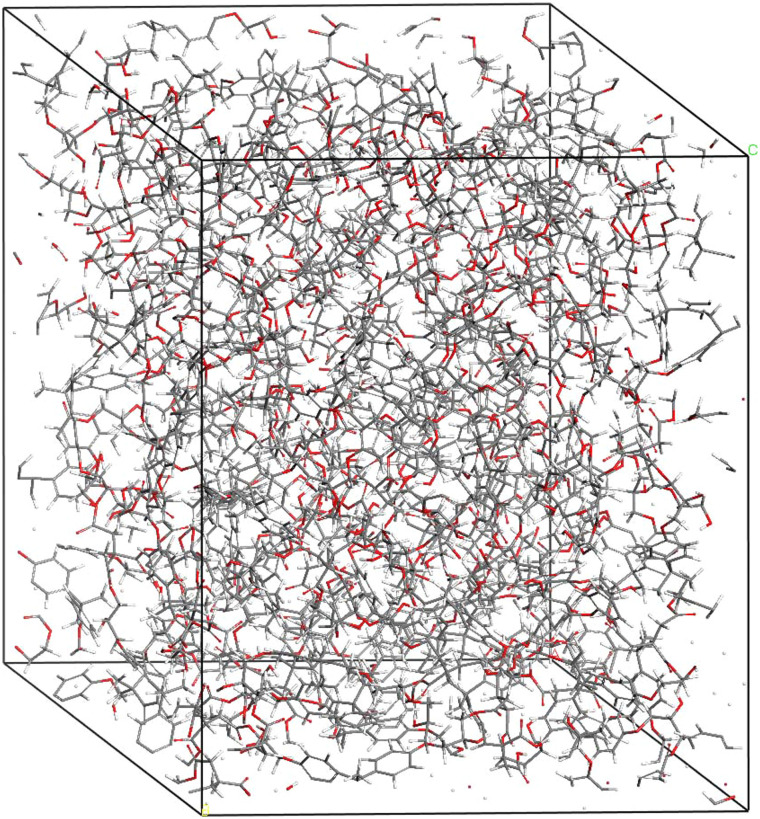
Epoxy model with complete crosslinking.

The crosslinking was performed as follows. Using the QT program, we bonded all pairs whose distance between the active site of the epoxy molecule and the active site of the crosslinking agent molecule was less than 5 Å. Then we performed geometric optimization, and carried out MD simulation using the NPT ensemble (500 K, 0.1 MPa) for 50 ps using the Forcite module.^42^ This process was repeated until no more binding pairs existed; the distance between the active site of the epoxy molecule and the active site of the crosslinking agent molecule was then increased by 0.5 Å and we completed the crosslinking by repeating the optimization and equilibration until the distance became 10 Å. For 99 epoxy molecules and 66 crosslinking agent molecules, the number of bonded pairs was 181, with a conversion of 91.41% and an average polymerization degree of 9.9. After the crosslinking was completed, the unbound active sites were deactivated and equilibrated again. The epoxy model is shown in Fig. 2. The density of the epoxy resin was calculated by averaging the density during the final 100 ps of the NPT simulation used for the equilibration. The calculated density of the epoxy resin is 1.197464 g cm^−3^, which has 0.49% relative error compared to the value in the previous work, 1.19158 g cm^−3^,^42^ which indicates that the applied PCFF force field is relatively suitable.

### 2.2 Modeling of coating

**Fig. 3:**
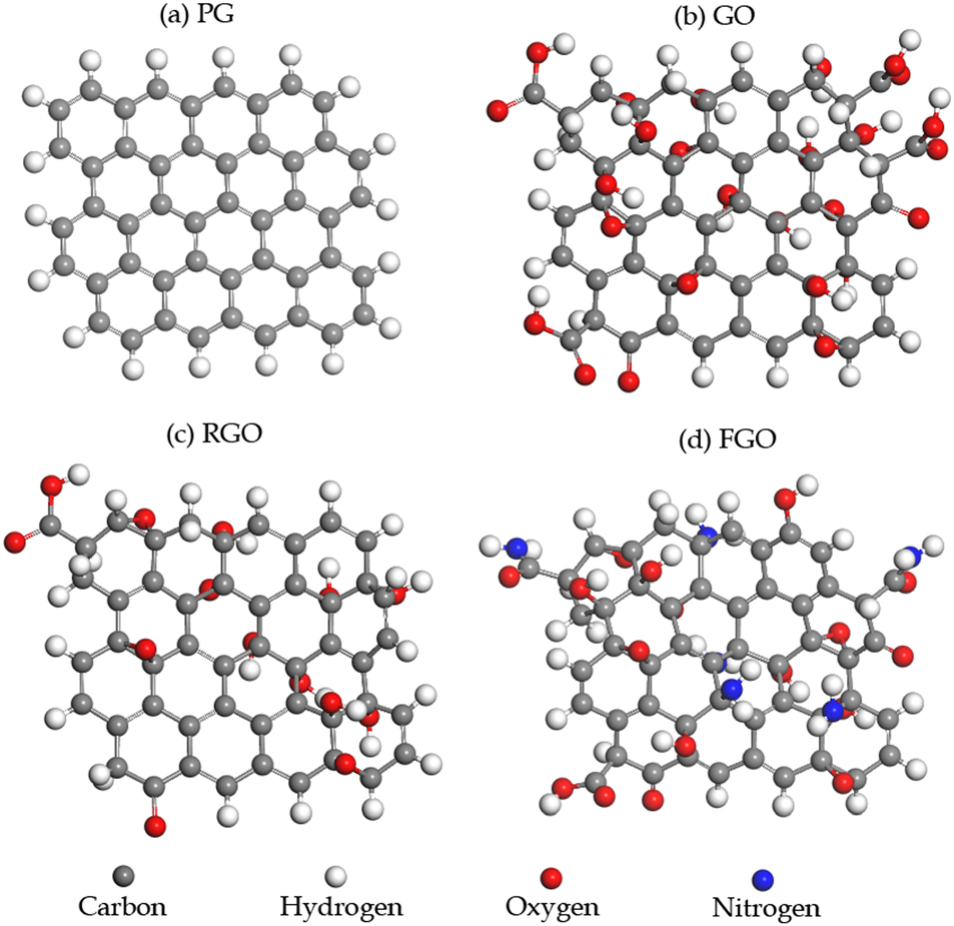
Molecular model of graphene derivatives (a: PG, b: GO, c: RGO, d: FGO).

The coating was modeled as a composite of graphene derivatives (PG, GO, RGO and FGO) on epoxy. The molecular structure of the graphene derivatives is shown in Fig. 3, and the detailed parameters are listed in Table 1.

**Table 1:** Modeling of the graphene derivatives

		Functional groups attached on the surface of the graphene								
Graphene type	Number of carbons in graphene	–O	=O	–OH	–COOH	–NH_2_	–C(=O)NH_2_	–H	Total number of atoms	C : O
PG	48	—	—	—	—	—	—	18	66	—
GO	52	6	2	10	4	—	—	18	110	2
RGO	49	4	1	7	1	—	—	20	91	3.5
FGO	51	4	2	6	1	4	2	17	110	3

**Fig. 4:**
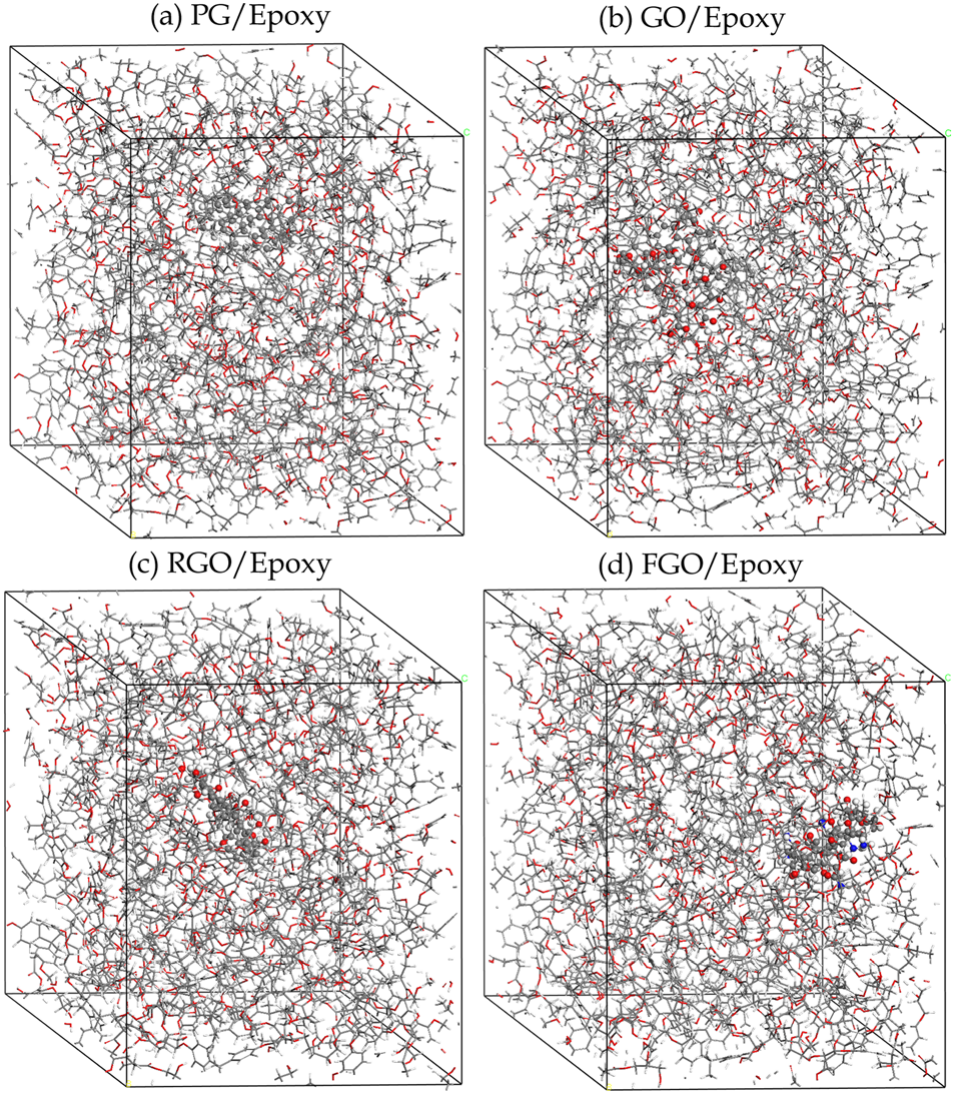
Coating model (a: PG/Epoxy, b: GO/Epoxy, c: RGO/Epoxy, d: FGO/Epoxy.

PG, GO, RGO and FGO were each inserted into the crosslinked epoxy model using the Amorphous Cell packing module,^43^ and the structures are shown in Fig. 4.

### 2.3 Modeling of corrosion medium

**Fig. 5:**
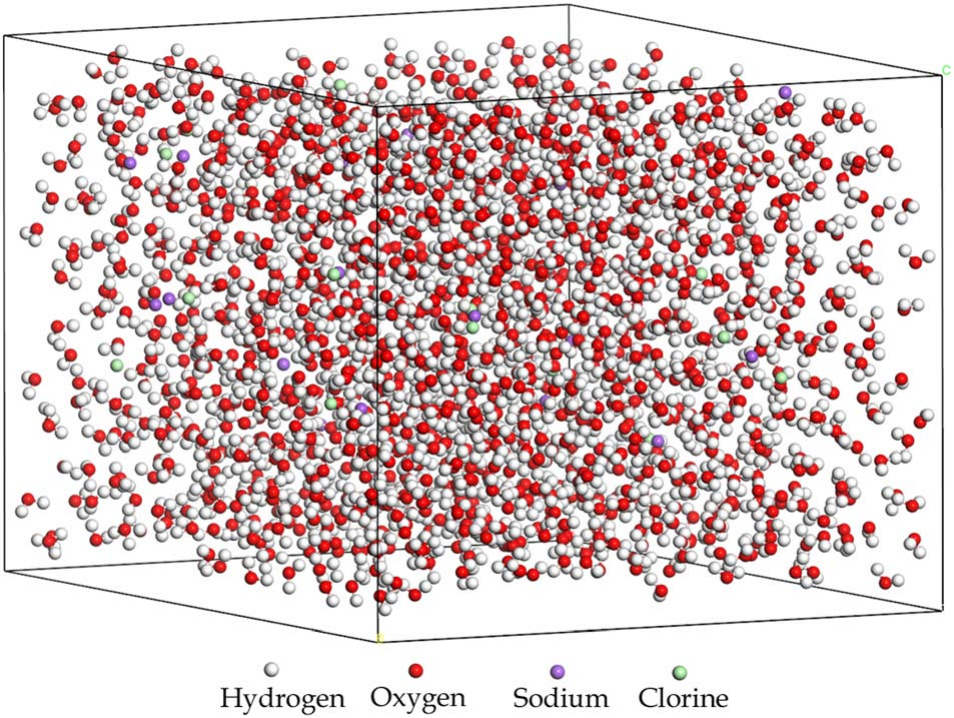
Corrosion medium (NaCl medium) model.

We set the NaCl concentration to 4% as the corrosion medium. The NaCl medium was modeled using the Amorphous Cell packing module with a cell size of 37.142445 × 37.142445 × 30.0 Å^3^, with water molecules, chloride anions and sodium cations chosen to have a NaCl concentration of 4% and a density of 1.1 g cm^−3^, and equilibrated. The structure of the completed corrosion medium (NaCl medium) is shown in Fig. 5.

During equilibration, the density of the NaCl medium was changed from 1.1 to 1.060323 g cm^−3^. This value reflects the experimental value of 1.024792 g cm^−3^ (ref. 44) with a relative error of 3.47%, which indicates that the force field is suitable.

### 2.4 Modeling and simulation methods of hybrid systems

The corrosion system is composed of the protected metal, the coating and the corrosion medium. The protected metal was modeled as Fe (110)^12^ and its size was 37.142445 × 37.142445 × 12.130672 Å^3^ (15 × 15 × 7). The coating was made of epoxy resin with or without graphene derivatives. In the simulation, the model was simulated in the same way for each model with five different types of coatings: iron/epoxy/NaCl medium (Model 1), iron/PG composite epoxy/NaCl medium (Model 2), iron/GO composite epoxy/NaCl medium (Model 3), iron/RGO composite epoxy/NaCl medium (Model 4), and iron/FGO composite epoxy/NaCl medium (Model 5). Parameters according to the models are listed in Table 2. To avoid diffusion of molecules to the vacuum region, we used a wall potential in the *z*-direction and periodic boundary conditions in the *x*- and *y*-directions.

**Table 2:** Atomistic data and modeling parameters used in MD simulations

	Parameters	Model 1	Model 2	Model 3	Model 4	Model 5
Iron	Surface	110	110	110	110	110
	Number of atoms	1350	1350	1350	1350	1350
Coating	Number of atoms	6205	6271	6315	6296	6315
	Degree of crosslinking (%)	91.41	91.41	91.41	91.41	91.41
	Density (g cm^−3^)	1.197464	1.200024	1.212835	1.19724	1.207543
	Graphene content (wt%)	—	1.294042	2.310438	1.819775	2.167522
NaCl medium	H_2_O : NaCl	1378 : 18	1378 : 18	1378 : 18	1378 : 18	1378 : 18
	Number of atoms	4170	4170	4170	4170	4170
	Density (g cm^−3^)	1.060323	1.060323	1.060323	1.060323	1.060323

First, the interaction between the graphene derivatives and epoxy resin was evaluated during the coating equilibration. Next, we combined the iron crystal model and coating model and evaluated the interaction between the coating and iron during equilibration. Finally, we combined iron, coating and NaCl medium models and performed MD simulation at 600 K for 10 ns using NVT ensembles to evaluate the coating properties and permeation coefficients. In practical systems, corrosion takes months to years due to the wear of the epoxy coating. In simulation, this timeframe is impossible due to the computational cost, so the simulation was carried out by increasing the temperature of the model system to 600 K to accelerate the time of corrosion. In all simulations, the timestep was 1 fs.

## 3 Results and discussion

### 3.1 Interaction between graphene derivatives and epoxy resins

The interaction energy between the graphene derivative and epoxy resin IE_g/e_ is calculated from the potential energy of the graphene derivative PE_g_, the potential energy of the epoxy resin PE_e_, and the total potential energy of the nanocomposite PE_g/e_.^39^

1 IE_g/e_ = PE_g/e_ − (PE_g_ + PE_e_)

**Fig. 6:**
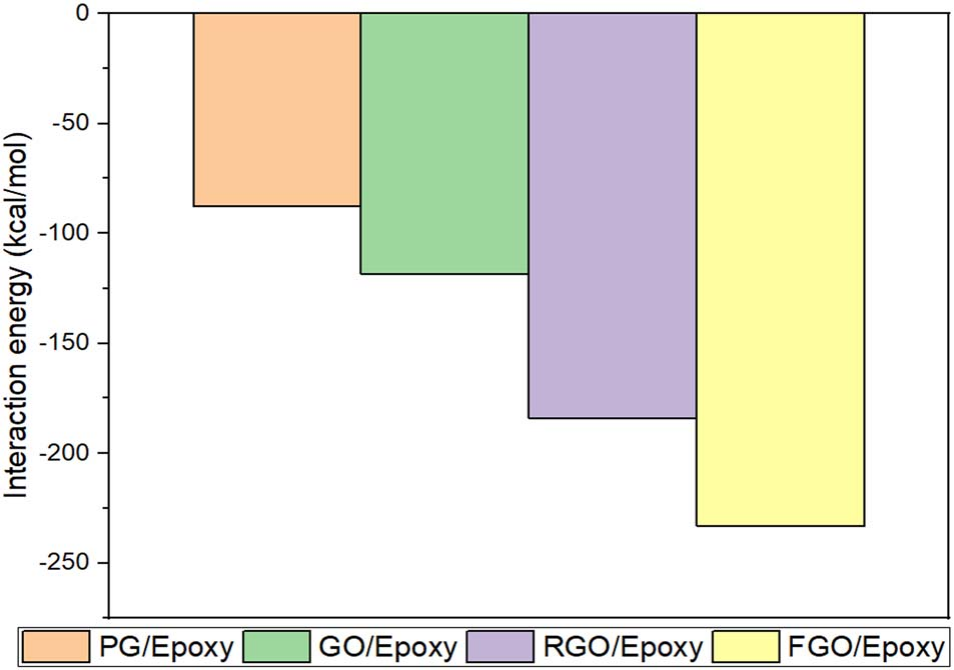
Interaction energy between graphene derivatives and epoxy resin.

After the coating equilibration, the PG/Epoxy, GO/Epoxy, RGO/Epoxy and FGO/Epoxy interaction energies are calculated according to eqn (1) and the results are shown in Fig. 6.

The lower the value of the interaction energy, the higher the interaction between the graphene derivative and epoxy resin. The interaction energy between PG and the epoxy resin is −87.5 kcal mol^−1^, but the interaction energy between GO and the epoxy resin is −118.3 kcal mol^−1^, the interaction energy with RGO is −184.3 kcal mol^−1^, and interaction energy between FGO and the epoxy resin is −233.0 kcal mol^−1^. This means that the interaction energies between GO, RGO, FGO and the epoxy resin are 135.2%, 210.6% and 266.2% higher than that between PG and the epoxy resin. This is due to the functional groups, such as –O–, –OH, –COOH and –NH_2_, on the surface of GO, RGO and FGO. The high polarity of these groups leads to high strength of interaction between the epoxy resin and graphene derivative *via* Coulomb interactions of these groups with epoxide –O–. The results of these simulations show the same trend as previous research^39^ that calculated the interaction energies for PG/Epoxy, GO/Epoxy and FGO/Epoxy.

The interaction energy between the graphene derivatives and epoxy resin may be considered as an indicator of how well the graphene derivative adheres to the epoxy resin in practice, *i.e.*, how well it disperses. In practice, RGO and FGO disperse well in epoxy resin, and PG has the worst dispersibility. Thus, the dispersibility of graphene in epoxy resin may be determined by surface functional groups; surface modification is carried out to improve the dispersibility of graphene, and the simulation results effectively illustrate the behavior of such surface functional groups of graphene.

### 3.2 Interaction between iron and the coating

The interaction energy IE_i/c_ between the coating and the iron is also calculated from the potential energy of the coating, PE_c_, the potential energy of the iron, PE_i_, and the total potential energy of the iron + coating, PE_i/c_, in the same way as the interaction energy between the graphene derivative and the epoxy resin.

Potential energy is composed of bond stretching, angle bending, dihedral torsion, out-of-plane potentials, cross-coupled terms describing the interactions between valence terms, and electrostatic and van der Waals potentials.^41,42^

2 IE_*i*/c_ = PE_*i*/c_ − (PE_*i*_ + PE_c_)

**Fig. 7:**
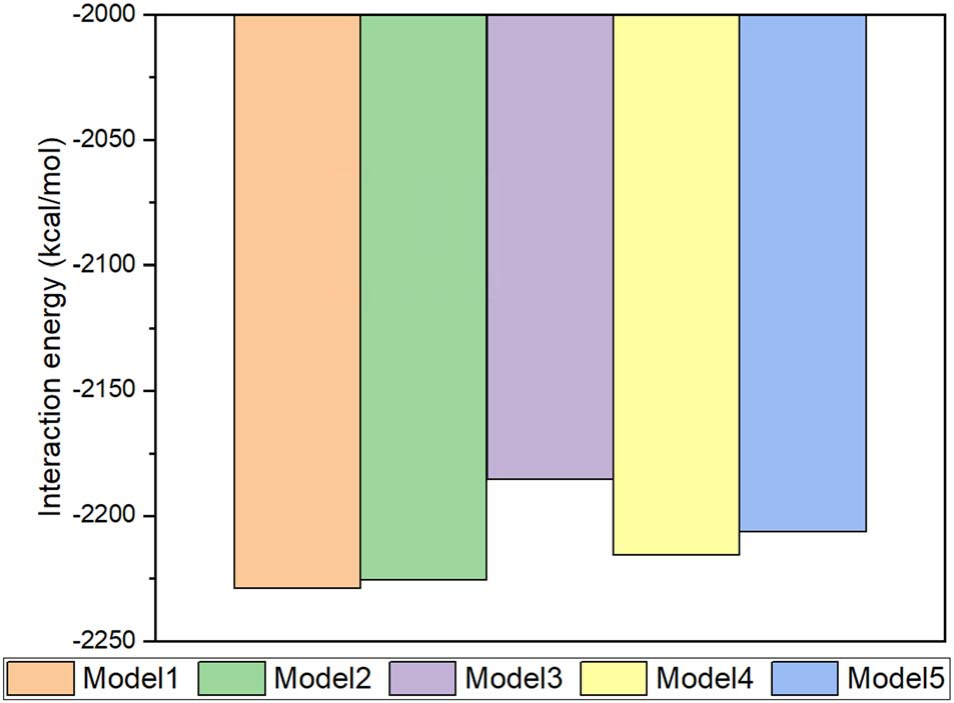
Interaction of the coating between the iron (Model 1: pure epoxy, Model 2: PG/Epoxy, Model 3: GO/Epoxy, Model 4: RGO/Epoxy, Model 5: FGO/Epoxy).

After the combining and equilibration of the iron crystal model and the coating model, the calculated interaction energy of the iron/coating according to eqn (2) is simulated for the five models of the coating, as shown in Fig. 7.

The interaction energy between the coating and the iron is the most prominent in Model 1 (epoxy-iron) at −2228.8 kcal mol^−1^, Model 2 (PG/Epoxy-iron) is second at −2225.7 kcal mol^−1^, Model 4 (RGO/Epoxy-iron) is third at −2215.7 kcal mol^−1^, then Model 5 (FGO/Epoxy) at −2206.4 kcal mol^−1^, and in Model 3 (GO/Epoxy-iron) the interaction is the lowest at −2185.3 kcal mol^−1^. The percentages of the values relative to Model 1 are 99.86% (Model 2), 99.41% (Model 4), 98.99% (Model 5) and 98.05% (Model 3), with no significant difference. This may indicate that the incorporation of a graphene derivative into epoxy resin slightly reduced the adhesion strength with iron.

**Fig. 8:**
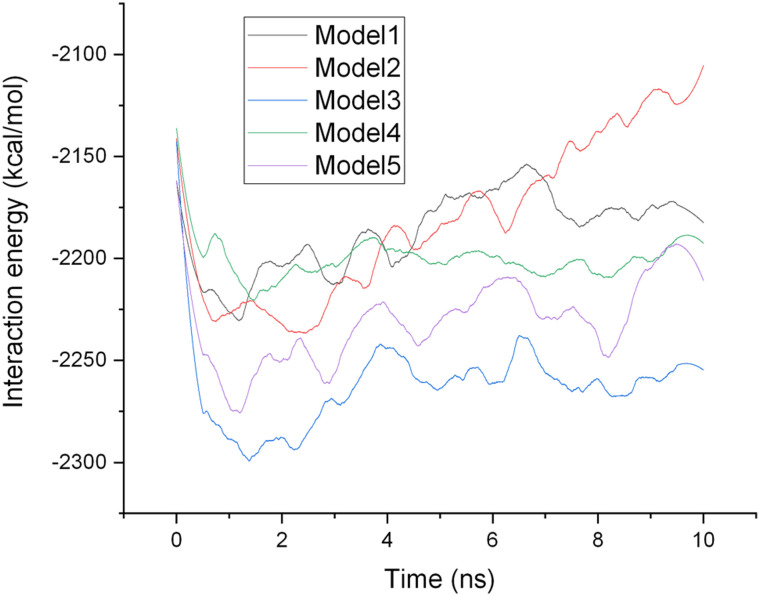
Interaction energy between the coating and the iron during the simulation (Model 1: pure epoxy, Model 2: PG/Epoxy, Model 3: GO/Epoxy, Model 4: RGO/Epoxy, Model 5: FGO/Epoxy).

To evaluate how the adhesion strength of the coating and the iron changes during corrosion, the interaction energy between the coating and the iron during the simulation was calculated, and the results are shown in Fig. 8. The change in the interaction energy (negative value) is as follows. It is the largest in Model 3 (GO/Epoxy-iron), as can be seen in the figure, and decreases in the order of Model 5 (FGO/Epoxy-iron), Model 4 (reduced GO/Epoxy-iron), Model 1 (epoxy-iron), and Model 2 (PG/Epoxy-iron). This change can be attributed to two reasons. First, the combining of graphene derivatives into epoxy resin affects the contraction stress of the coating, which leads to an increase in density and internal stress, resulting in an increase in adhesion strength. This can also be seen from the density of the coating models (Table 2). Secondly, it is attributed to the influence of water molecules that have permeated into the coating during corrosion. The presence of water molecules reduces the interfacial adhesion due to excessive interaction *via* chemical, polar and hydrogen bonds. For both reasons, the interaction energy between the coating and iron changes during corrosion, and the density of the coatings and the quantitative relationship of water molecules that have permeated into the coating during corrosion effectively explain the magnitude of this change in interaction energy. Thus, Model 3 with the largest density and the smallest number of permeating water molecules has the highest strength of interaction, and the other models also determine the strength-size relationship of the interaction in proportion to this.

### 3.3 Wear properties of coatings

An organic coating on the surface of a substrate is destroyed by the influence of corrosion medium after a certain time. As the coating breaks, it will not act as a protective layer, thus accelerating the corrosion process of the metal substrate. Therefore, simulating the wear properties of coatings during the simulation is of considerable significance in the study of corrosion of metal protected by coatings.

**Fig. 9:**
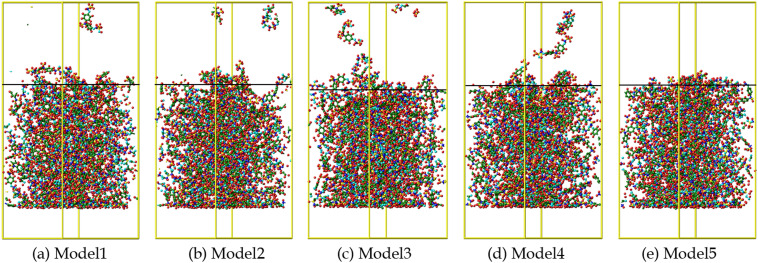
Structural state of the corrosion layer after simulation.

In this paper, the wear properties of the coatings during corrosion were evaluated by calculating the number of atoms that constitute the epoxy resin in the coating region before simulation, and the number leaving the coating region near the end of simulation. After the simulation of the corrosion process, the states of the coating are shown in Fig. 9.

**Fig. 10:**
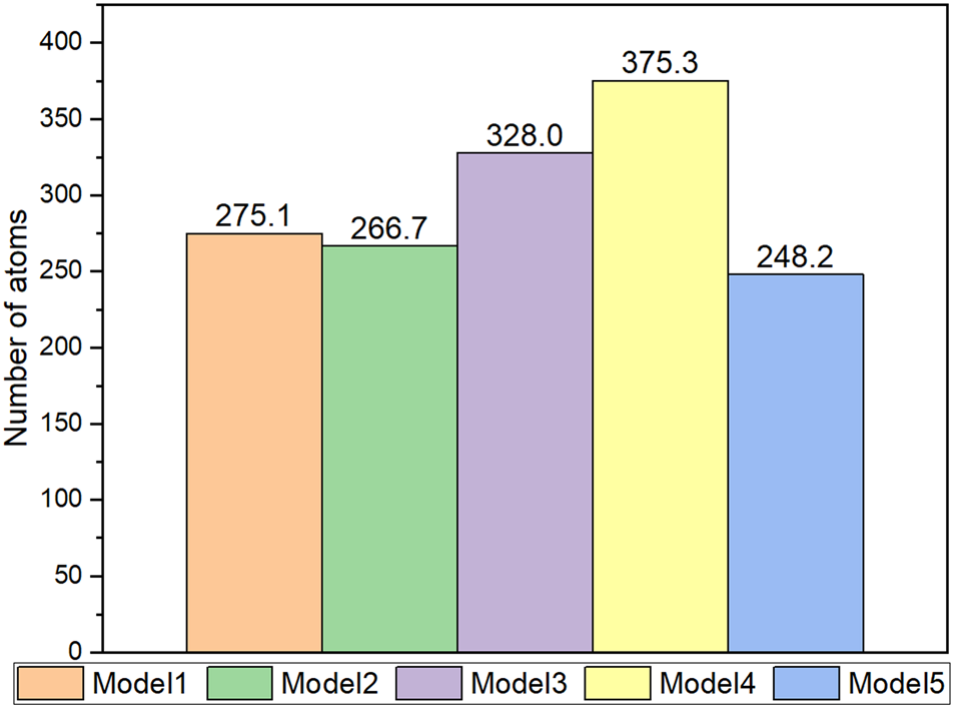
Structure of the etched layer beyond the coating region after simulation (Model 1: pure epoxy, Model 2: PG/Epoxy, Model 3: GO/Epoxy, Model 4: RGO/Epoxy, Model 5: FGO/Epoxy).

The black horizontal line in Fig. 9 represents the boundary of the coating section before simulation. As shown in the figure, some of the non-crosslinked epoxy monomers easily leave the coating region. To quantify the degree of wear in the coating, the number of atoms outside the coating region during the last 100 ps of simulation was averaged, and the results are shown in Fig. 10. As shown in Fig. 10, the number of atoms leaving the coating region is 275.1 in Model 1 (epoxy-iron), 4.43% of the number of its coating atoms, 266.7 in Model 2 (PG/Epoxy-iron), 4.25% of the number of its coating atoms, 328.0 in Model 3 (GO/Epoxy-iron), 5.19% of the number of its coating atoms, and 375.3 in Model 4 (RGO/Epoxy-iron), 5.96% of the number of its coating atoms, and 248.2 in Model 5 (FGO/Epoxy-iron), 3.93% of the number of its coating atoms. The results of these calculations show that the incorporation of graphene or FGO into the epoxy coating in a NaCl medium environment leads to a stronger bond, whereas the incorporation of GO or RGO leads to a weaker bond. The results are comparable to the results of the interaction energy between the epoxy resin and the graphene derivatives with or without NaCl medium (Section 3.1), which may be attributed to the influence of the NaCl medium environment.

### 3.4 Permeation coefficient

Corrosion in the coated metal is caused by the formation of an electrochemical double layer, which is driven by the water permeation between the metal substrate and the organic coating, through the coating. Therefore, the corrosion properties of organically coated metals are often evaluated *via* the permeation coefficient *P*, which indicates the permeation of the coating. The permeation coefficient is defined as the product of the water solubility and diffusion coefficient in the coating layer and the specific mass of water.^45^

The water solubility *S* (kg cm^−3^) may be calculated from the number of water molecules that have permeated into the coating layer and the volume of the coating after the simulation.3
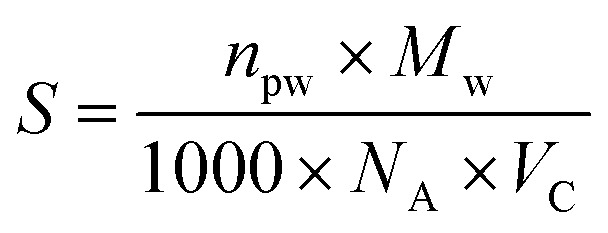
where *n*_pw_ is the number of water molecules that have permeated into the coating; *M*_w_ represents the molecular weight of water molecules (18.0152 g mol^−1^); *N*_A_ represents the Avogadro constant (6.0221415 × 10^23^/mol); and *V*_C_ represents the volume of the coating (cm^3^). The diffusion coefficient of water *D*_w_ (m^2^ s^−1^) is calculated from the mean square displacement (MSD) using the Einstein diffusion equation.^19^4
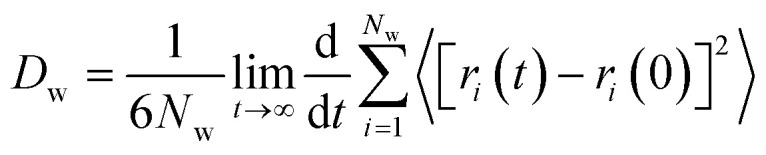

**Fig. 11:**
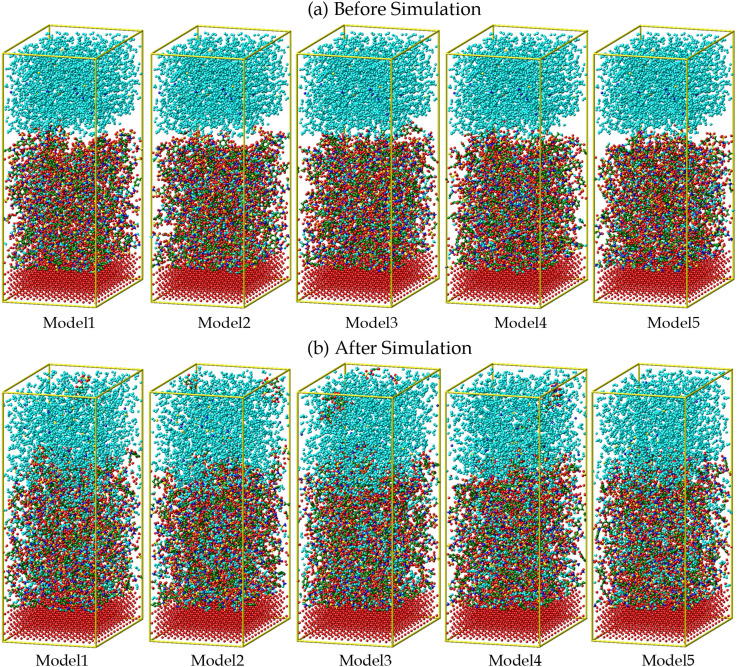
Model of the system before and after simulation (Model 1: pure epoxy, Model 2: PG/Epoxy, Model 3: GO/Epoxy, Model 4: RGO/Epoxy, Model 5: FGO/Epoxy).

5 MSD = 〈|*r*_*i*_(*t*) − *r*_*i*_(0)|^2^〉

where *N*_w_ represents the number of water molecules, *r*(0) the initial coordinate of the water molecules, and *r*(*t*) the coordinate of the water molecules at time *t*. The specific mass of water *p* (kg m^−2^) may be calculated from the number of water molecules and the area of the coating.6
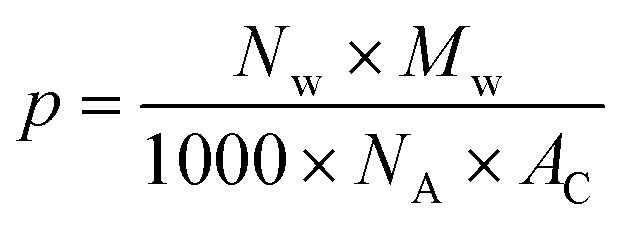
where *A*_C_ (m^2^) represents the area of the coating. The permeation coefficient of the coating is calculated after the simulation of the corrosion process is completed. The model structure before and after the simulation of the whole system is shown in Fig. 11.

The number of water molecules that had permeated into the coating, the solubility, diffusion coefficient, specific mass and permeation coefficient obtained according to eqn (3)–(6), and the relative amount of permeation compared to Model 1 are given in Table 3. The number of water molecules that had permeated into the coating decreased significantly for the coatings made of nanocomposites, with the smallest for GO/Epoxy coating, then a small number for the FGO/Epoxy coating, and similar for the PG/Epoxy coating and RGO/Epoxy coating. The diffusion coefficient was slightly larger in the graphene/epoxy coating compared to the epoxy coating and smaller in coatings made of other nanocomposites. This may be attributed to the lowest interaction energy between graphene and epoxy in the graphene/epoxy coating.

**Table 3:** Number of permeating water molecules, solubility, diffusion coefficient, specific mass and permeability of each model

	Model 1	Model 2	Model 3	Model 4	Model 5
Number of permeating water molecules	290.6	271.3	243.2	270.8	252.7
Solubility (kg cm^−3^)	1.0045 × 10^−4^	9.3939 × 10^−5^	8.6762 × 10^−5^	9.5217 × 10^−5^	8.8025 × 10^−5^
Diffusion coefficient (m^2^ s^−1^)	1.6500 × 10^−8^	1.6833 × 10^−8^	1.4833 × 10^−8^	1.4667 × 10^−8^	1.5500 × 10^−8^
Specific mass (kg m^−2^)	3.1690 × 10^−6^	3.1690 × 10^−6^	3.1690 × 10^−6^	3.1690 × 10^−6^	3.1690 × 10^−6^
Permeation coefficient	5.2526 × 10^−18^	5.0111 × 10^−18^	4.0784 × 10^−18^	4.4255 × 10^−18^	4.3237 × 10^−18^
Relative amount of permeation	100	95.4	77.6	94.3	82.3

**Fig. 12:**
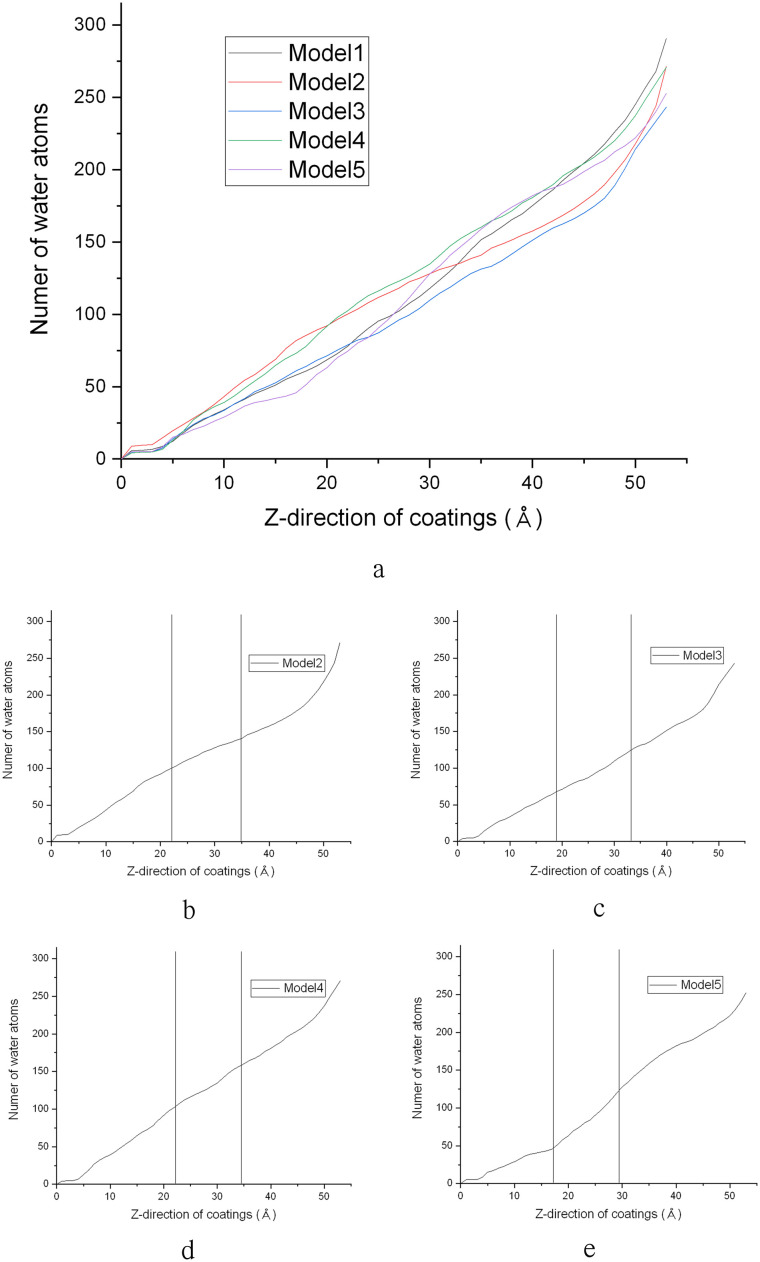
Water distribution in the coatings after simulation.

Finally, the permeation coefficient is the smallest for GO/Epoxy coating. In other words, the best anticorrosive properties may be inferred. It can be seen that the PG/Epoxy coating has a higher permeation coefficient compared to the other nanocomposites, which is attributed to the action of the surface functional groups of graphene. To evaluate the permeation rate of water into the coating in detail, the cumulative distribution of the number of permeating water molecules per depth of the coating was obtained after simulation, as shown in Fig. 12. Fig. 12(a) shows all models in one figure, and Fig. 12(b)–(e) show the graphene derivative/epoxy models separately. In Fig. 12(b)–(e), the graphene derivatives are located between two vertical lines in the *z*-direction. As shown in Fig. 12(b)–(e), the amount of permeation decreased under graphene derivatives. This shows us that graphene derivatives act as a barrier against the penetration of water molecules in the coating, and the range of their action is 10–15 Å beyond the graphene derivatives.

Electrochemical corrosion occurs when electrolyte ions are present along with water molecules. So, the number of sodium cations and chloride anions that permeate the coating was calculated. The number of electrolyte ions that permeated into the coating was calculated by averaging the number of permeating ions during the last 100 ps of the simulation, and the results are shown in Table 4.

**Table 4:** Number of ions that permeated into the coating during molecular dynamics simulations

	Model 1	Model 2	Model 3	Model 4	Model 5
Na^+^	0.568	0.473	0.276	0.321	0.295
Cl^−^	0.404	0.335	0.159	0.213	0.156

As shown in Table 4, the permeation properties of the electrolyte ions also show the same trend as the water molecules. Thus, the incorporation of a graphene derivative into epoxy can effectively enhance the anticorrosion resistance, again confirming that the GO/Epoxy coating has the best corrosion resistance among them. The reason why the GO/Epoxy coating has the highest corrosion resistance can be considered as follows. The surface of GO contains more oxygen-containing groups than other graphene derivatives, which form a partially negatively charged electron cloud on the surface of GO, thus blocking corrosive agents such as water to prevent redox reactions. Generally, the GO surface is not smooth, so oxygen-containing functional groups on the surface fill up the pores of the surface. However, during the reduction or amine functionalization process of GO, the surface of the graphene is flattened by the conversion of sp^3^ hybridization to sp^2^, allowing the pores to be exposed to the surface. Thus, it may have an adverse effect on the prevention of corrosion. Nevertheless, GO has insulating properties due to its lower conductivity than other graphene derivatives, which prevents corrosion, and reducing GO or amine functionalization may promote corrosion due to the increased conductivity. The corrosion properties of a graphene derivative/glycine coating have been studied *via* electrochemical (CV, LSV, EIS) methods, and the corrosion resistance was reported to be the best for the GO/glycine coating.^29^ Meanwhile, the corrosion properties of GO and RGO/epoxy composite coatings were investigated *via* the EIS method, and the composite epoxy coating with GO was reported to be superior to epoxy resin with RGO.^46^ The above experimental results are in agreement with our simulation results.

## 4 Conclusions

In this paper, we modeled corrosion systems composed of iron/graphene derivative/epoxy/NaCl medium and evaluated the effect of graphene derivatives in the epoxy coating on the corrosion of iron, using MD.

Graphene derivatives were modeled as PG, GO, RGO, and FGO, and the number of hexagonal rings in each graphene derivative was set equally. The interaction energy between the graphene derivatives and epoxy resin in the nanocomposites increases in the order of PG (−87.5 kcal mol^−1^), GO (−118.3 kcal mol^−1^), RGO (−184.3 kcal mol^−1^), FGO (−233.0 kcal mol^−1^).

During the simulation, the interaction energy between the nanocomposites and iron was greatest in GO/Epoxy, and it is decreased in the order of FGO/Epoxy, RGO/Epoxy, pure epoxy, and PG/Epoxy. In the simulation, the degree of wear in the coating with PG and FGO was smaller than in the epoxy coating, but it was larger in the coating with GO and RGO.

The permeation coefficient of the coating was reduced to 95.4% in the PG/Epoxy coating, 77.6% in the GO/Epoxy coating, 84.3% in the RGO/Epoxy coating and 82.3% in the FGO/Epoxy coating compared to the pure epoxy coating. The permeation properties of electrolyte ions into the coating also showed a similar tendency to water molecules. The simulation results show that the incorporation of graphene into pure epoxy resin may improve its corrosion resistance and oxygen-containing graphene derivatives were preferable to PG. Our results are useful for understanding the reinforcement mechanism of graphene derivatives in graphene/epoxy composites and designing graphene coating materials with better corrosion resistance.

## Author contributions

Jae-Hyok Ju developed the original project and drafted the first manuscript. Ryong-Chol Ri performed the calculations and post-processing. Il-Chol Ju, Chol-Nam Song, Ryong-Hyon Gong and Jae-Gwang Ri assisted with the calculations and contributed to useful discussions.

## Conflicts of interest

There are no conflicts to declare.

## Data Availability

The data that support the findings of this study are available from the corresponding authors upon reasonable request.
